# Supplementary material for: Exosome Liberation by Human Neutrophils under L-Amino Acid Oxidase of Calloselasma rhodostoma Venom Action
Source: Toxins (Basel). 2023 Oct 25;15(11):625. doi: 10.3390/toxins15110625 (PMC10674320; doi:10.3390/toxins15110625)
Supplement: Supplementary file 1 [file toxins-15-00625-s001.zip › toxins-2612832- supplymentary Material S2.pdf]

Article

Exosome Liberation by Human Neutrophils under L-Amino Acid Oxidase of *Calloselasma rhodostoma* Venom Action

Suzanne N. Serrath [1], Adriana S. Pontes. [1], Mauro V. Paloschi [1], Milena D. S. Silva. [1], Jéssica A. Lopes [1],
Charles N. Boeno [1], Carolina P. Silva [1], Hallison M. Santana [1], Daniel G. Cardozo [1], Andrey V. E. Ugarte [1],
João G. S. Magalhães [1], Larissa F. Cruz [1], Sulamita S. Setubal [1], Andreimar M. Soares [2], Bruna Cavecci-Mendonça [3], Lucilene D. Santos [3,4] and Juliana P. Zuliani. [1,5,*]

Table S1. Unique proteins present in the experimental LAAO group as compared to the Control group through proteomic analysis.

Access Code	Description of Protein
P17661	Desmin
P48594	Serpin B4
P01008	Antithrombin-III
A0A0A0MTS7	Titin
A6XMV9	Protease serine 2 preproprotein
P00338	L-lactate dehydrogenase A chain
P07195	L-lactate dehydrogenase B chain
P63104	14-3-3 protein zeta/delta
P62258	14-3-3 protein epsilon
P31946	14-3-3 protein beta/alpha
Q04917	14-3-3 protein eta
P61981	14-3-3 protein gamma
P27348	14-3-3 protein theta
U3KQK0	Histone H2B
P52179	Myomesin-1
K7ES00	Histone H3.3 Fragment
H7BZJ8	Zinc-alpha-2-glycoprotein
P21796	Voltage-dependent anion-selective channel protein 1
P07451	Carbonic anhydrase 3
P01042	Kininogen-1
A0A087X1N7	Nebulin
P01834	Immunoglobulin kappa constant
Q99798	Aconitate hydratase, mitochondrial
P32119	Peroxiredoxin-2 OS=Homo sapiens
A0A494C165	Xaa-Pro dipeptidase (Fragment)
Q86TD4	Sarcalumenin OS=Homo sapiens
P05089	Arginase-1 OS=Homo sapiens
P36957	Dihydrolipoyllysine-residue succinyltransferase component of 2-oxoglutarate dehydrogenase complex, mitochondrial

Table S2. Unique proteins present in the experimental Control group as compared to the LAAO group through proteomic analysis.

Access Code	Description of Protein
P35580	Myosin-10
P08670	Vimentin
P02675	Fibrinogen beta chain
P11678	Eosinophil peroxidase
P02679	Fibrinogen gamma chain
P07996	Thrombospondin-1
P35443	Thrombospondin-4
P35442	Thrombospondin-2
P11215	Integrin alpha-M
Q9H4B7	Tubulin beta-1
A0A0B4J269	G_Protein_Recep_F1_2 domain-containing protein
P13807	Glycogen [starch] synthase, muscle
H0Y300	Haptoglobin
P61158	Actin-related protein 3
P46976	Glycogenin-1
P52790	Hexokinase-3
O00160	Unconventional myosin-If
P01009	Alpha-1-antitrypsin
P61626	Lysozyme C
P30153	Serine/threonine-protein phosphatase 2A 65 kDa regulatory subunit A alpha isoform
Q01432	AMP deaminase 3
E9PKC5	AMP deaminase
P08133	Annexin A6
E5RK69	Annexin
P29350	Tyrosine-protein phosphatase non-receptor type 6
O75083	WD repeat-containing protein 1
P15153	Ras-related C3 botulinum toxin substrate 2
P04746	Pancreatic alpha-amylase
P53621	Coatomer subunit alpha
P00488	Coagulation factor XIII A chain
P08697	Alpha-2-antiplasmin
O75955	Flotillin-1
P17858	ATP-dependent 6-phosphofructokinase, liver type
F5H2F4	C-1-tetrahydrofolate synthase, cytoplasmic
A0A0A0MRJ7	Coagulation factor V
O95466	Formin-like protein 1
Q12905	Interleukin enhancer-binding factor 2
P01040	Cystatin-A
B0QZ18	Copine-1
P50991	T-complex protein 1 subunit delta
A0A024R571	EH domain-containing protein 1
Q9NZN3	EH domain-containing protein 3
Q14166	Tubulin--tyrosine ligase-like protein 12

Q99536	Synaptic vesicle membrane protein VAT-1 homolog
P19827	Inter-alpha-trypsin inhibitor heavy chain H1
P43490	Nicotinamide phosphoribosyltransferase
P26640	Valine-tRNA ligase
A6NHR9	Structural maintenance of chromosomes flexible hinge domain-containing protein 1
Q16851	UTP-glucose-1-phosphate uridylyltransferase
P00734	Prothrombin
P07384	Calpain-1 catalytic subunit
P61160	Actin-related protein 2
A0A0A0MTH3	Integrin-linked protein kinase
O14556	Glyceraldehyde-3-phosphate dehydrogenase
P51884	Lumican
P19878	Neutrophil cytosol factor 2
Q14624	Inter-alpha-trypsin inhibitor heavy chain H4
P12109	Collagen alpha-1(VI) chain
P02647	Apolipoprotein A-I
H7C1H2	26S proteasome non-ATPase regulatory subunit 2
A8MVU1	Putative neutrophil cytosol factor 1C
P17213	Bactericidal permeability-increasing protein
P50995	Annexin A11
Q6UX06	Olfactomedin-4
Q99832	T-complex protein 1 subunit eta
P53396	ATP-citrate synthase
Q5D862	Filaggrin-2
P26022	Pentraxin-related protein PTX3
Q9Y2J8	Protein-arginine deiminase type-2
Q13201	Multimerin-1
Q14764	Major vault protein
P08575	Receptor-type tyrosine-protein phosphatase C
P01031	Complement C5
P08514	Integrin alpha-IIb
P22894	Neutrophil collagenase
P18206	Vinculin
P00747	Plasminogen
P26641	Elongation factor 1-gamma
P35573	Glycogen debranching enzyme
P02671	Fibrinogen alpha chain
P31944	Caspase-14

Table S3. Unique proteins present in the experimental LAAO group when compared to the PMA group through proteomic analysis.

Access Code	Description of Protein
P06744	Glucose-6-phosphate isomerase
P52209	6-phosphogluconate dehydrogenase
K7EMN2	6-phosphogluconate dehydrogenase
Q02413	Desmoglein-1
P29401	Transketolase
P54296	Myomesin-2
P0CG48	Polyubiquitin-C
P62258	14-3-3 protein epsilon
K7ES00	Histone H3.3
Q96P63	Serpin B12
P07355	Annexin A2
P00338	L-lactate dehydrogenase A chain
A6XMV9	Protease serine 2 preproprotein
U3KQK0	Histone H2B
P52179	Myomesin-1
P50395	Rab GDP dissociation inhibitor beta O
P31151	Protein S100-A7
Q01518	Adenylyl cyclase-associated protein 1
Q14315	Filamin-C
P31146	Coronin-1A
Q9Y490	Talin-1
P20929	Nebulin
A0A087X1N7	Nebulin
P09960	Leukotriene A-4 hydrolase
D6RF35	Vitamin D-binding protein
P48594	Serpin B4
P55072	Transitional endoplasmic reticulum ATPase
P20472	Parvalbumin alpha
J3KNB4	Cathelicidin antimicrobial peptide OS=Homo sapiens
P01042	Kininogen-1
P21796	Voltage-dependent anion-selective channel protein 1
P02751	Fibronectin
Q99798	Aconitate hydratase, mitochondrial
P01834	Immunoglobulin kappa constant
P36957	Dihydrolipoyllysine-residue succinyltransferase component of 2-oxoglutarate dehydrogenase complex, mitochondrial
P32119	Peroxiredoxin-2
P11413	Glucose-6-phosphate 1-dehydrogenase
P05089	Arginase-1

Table S4. Unique proteins present in the experimental PMA group when compared to the LAAO group through proteomic analysis.

Access Code	Description of Protein
H3BTW4	Sarcoplasmic/endoplasmic reticulum calcium ATPase 1
Q9H4B7	Tubulin beta-1 chain
P61626	Lysozyme C
A0A286YFJ8	Immunoglobulin heavy constant gamma 4
A0A0G2JPA8	Alpha-2-antiplasmin
P01009	Alpha-1-antitrypsin
P52790	Hexokinase-3
P00505	Aspartate aminotransferase, mitochondrial
O14556	Glyceraldehyde-3-phosphate dehydrogenase
P51884	Lumican
Q14624	Inter-alpha-trypsin inhibitor heavy chain H4
P00734	Prothrombin
Q6UX06	Olfactomedin-4
P01031	Complement C5
Q562E7	WD repeat-containing protein 81
P13639	Elongation factor 2
P22894	Neutrophil collagenase
P18206	Vinculin

Table S5. Unique proteins present in the experimental PMA group when compared to the Control group through proteomic analysis.

Access Code	Description of Protein
P17661	Desmin
P01008	Antithrombin-III
A0A0A0MTS7	Titin
P00505	Aspartate aminotransferase, mitochondrial
P07451	Carbonic anhydrase 3
P13639	Elongation factor 2
A0A494C165	Xaa-Pro dipeptidase (Fragment)
Q562E7	WD repeat-containing protein 81
Q86TD4	Sarcalumenin

Table S6. Unique proteins present in the experimental Control group when compared to the PMA group through proteomic analysis.

Access Code	Description of Protein
P35580	Myosin-10
P08670	Vimentin
Q9Y490	Talin-1
P29401	Transketolase
A0A0B4J1R6	Transketolase
P02675	Fibrinogen beta chain
P11678	Eosinophil peroxidase
Q01518	Adenylyl cyclase-associated protein 1
P02679	Fibrinogen gamma chain
P07996	Thrombospondin-1
P35443	Thrombospondin-4
P49747	Cartilage oligomeric matrix protein
P35442	Thrombospondin-2
P11215	Integrin alpha-M
P06744	Glucose-6-phosphate isomerase
P52209	6-phosphogluconate dehydrogenase, decarboxylating
P13807	Glycogen [starch] synthase, muscle
P31146	Coronin-1A
H0Y300	Haptoglobin
P61158	Actin-related protein 3
P46976	Glycogenin-1
O00160	Unconventional myosin-If
P30153	Serine/threonine-protein phosphatase 2A 65 kDa regulatory subunit A alpha isoform
Q01432	AMP deaminase 3
E9PKC5	AMP deaminase
P08133	Annexin A6
E5RK69	Annexin
P29350	Tyrosine-protein phosphatase non-receptor type 6
O75083	WD repeat-containing protein 1
P15153	Ras-related C3 botulinum toxin substrate 2
P04746	Pancreatic alpha-amylase
P53621	Coatomer subunit alpha
P00488	Coagulation factor XIII A chain
P0CG48	Polyubiquitin-C
P50395	Rab GDP dissociation inhibitor beta
O75955	Flotillin-1
P31151	Protein S100-A7
P17858	ATP-dependent 6-phosphofructokinase, liver type
Q02413	Desmoglein-1
F5H2F4	C-1-tetrahydrofolate synthase, cytoplasmic
A0A0A0MRJ7	Coagulation factor V
O95466	Formin-like protein 1
Q12905	Interleukin enhancer-binding factor 2
P01040	Cystatin-A

A6NMY6	Putative annexin A2-like protein
B0QZ18	Copine-1
P50991	T-complex protein 1 subunit delta
A0A024R571	EH domain-containing protein 1
Q9NZN3	EH domain-containing protein 3
Q14166	Tubulin--tyrosine ligase-like protein 12
Q99536	Synaptic vesicle membrane protein VAT-1 homolog
P19827	Inter-alpha-trypsin inhibitor heavy chain H1
P54296	Myomesin-2 OS=Homo sapiens
P43490	Nicotinamide phosphoribosyltransferase
Q96P63	Serpin B12
P26640	Valine--tRNA ligase
A6NHR9	Structural maintenance of chromosomes flexible hinge domain-containing protein 1
Q16851	UTP--glucose-1-phosphate uridylyltransferase
P07384	Calpain-1 catalytic subunit
P61160	Actin-related protein 2
A0A0A0MTH3	Integrin-linked protein kinase
P09960	Leukotriene A-4 hydrolase
P02647	Apolipoprotein A-I
P55072	Transitional endoplasmic reticulum ATPase
D6RF35	Vitamin D-binding protein
P19878	Neutrophil cytosol factor 2
P02751	Fibronectin
P12109	Collagen alpha-1(VI) chain
H7C1H2	26S proteasome non-ATPase regulatory subunit 2 (Fragment)
J3KNB4	Cathelicidin antimicrobial peptide
P20472	Parvalbumin alpha
A8MVU1	Putative neutrophil cytosol factor 1C
P17213	Bactericidal permeability-increasing protein
P11413	Glucose-6-phosphate 1-dehydrogenase
P50995	Annexin A11
Q99832	T-complex protein 1 subunit eta
P53396	ATP-citrate synthase
Q5D862	Filaggrin-2
P26022	Pentraxin-related protein PTX3
Q9Y2J8	Protein-arginine deiminase type-2
Q13201	Multimerin-1
Q14764	Major vault protein
P08575	Receptor-type tyrosine-protein phosphatase C
P08514	Integrin alpha-IIb
P00747	Plasminogen
P35573	Glycogen debranching enzyme
P02671	Fibrinogen alpha chain
P31944	Caspase-14
P26641	Elongation factor 1-gamma

Attached is the electrophoretic profile of exosome samples according to figure 1A.

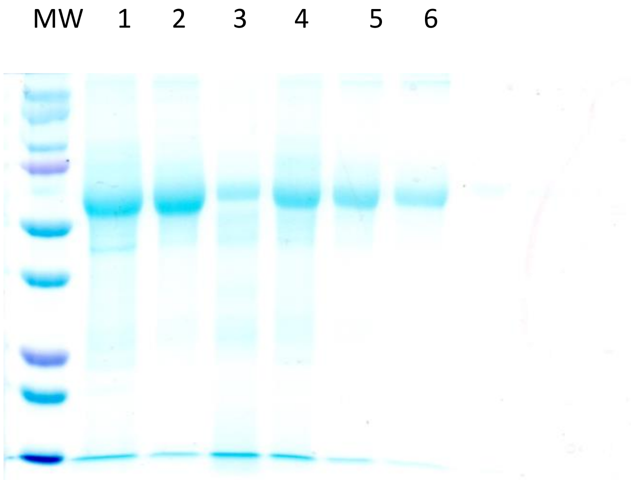

Subtitle: MW: molecular weight, 1: negative control (RPMI) N1, 2: positive control (PMA) N2, 3: Cr-LAAO N3, 4: negative control (RMPI) N1, 5: positive control (PMA) N2, 6: Cr-LAAO N3.

Attached, image of CD81 antibody immunoreactivity, according to figure 2E.

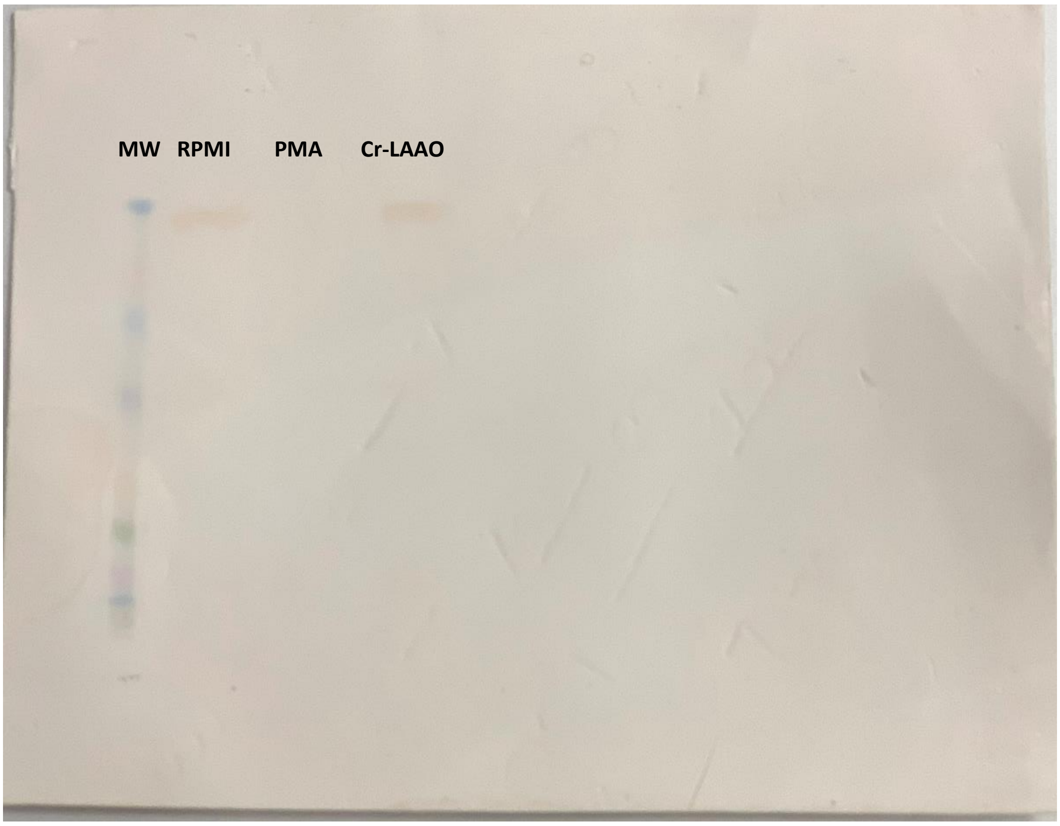

Subtitle: MW; molecular weight, negative control (RMPI), positive control (PMA), Cr-LAAO.

Attached, image of CD63 antibody immunoreactivity, according to figure 2E.

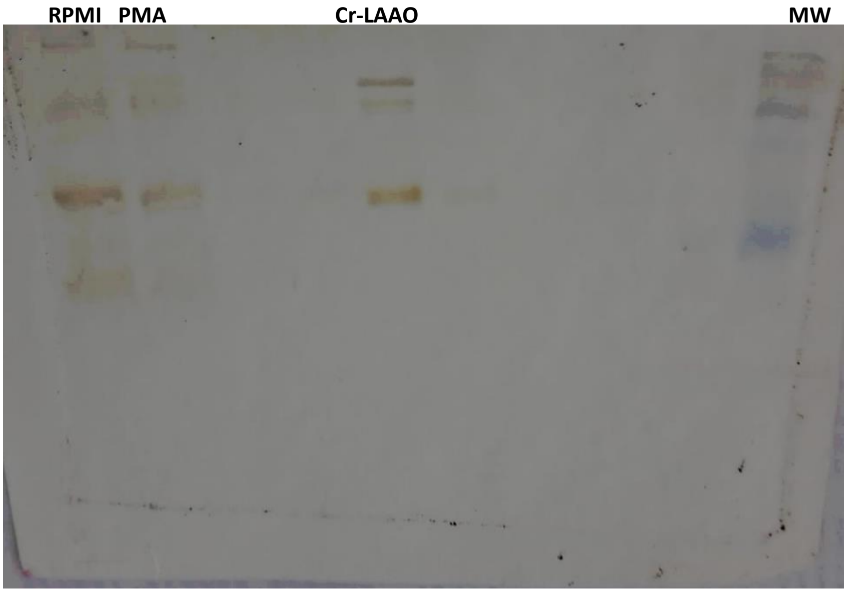

Subtitle: positive control (RPMI), positive control (PMA), Cr-LAAO, molecular weight (MW).
